# Curve-Fitting Algorithm for the Inspection of Subtle Feature Lines on Automotive Outer Panels

**DOI:** 10.3390/ma15186323

**Published:** 2022-09-12

**Authors:** Chang-Whan Lee, Yun-Chan Chung

**Affiliations:** Department of Mechanical System Design Engineering, Seoul National University of Science and Technology, Seoul 01811, Korea

**Keywords:** subtle feature line, sheet metal forming, automotive outer panel, RANSAC, curve fitting

## Abstract

Feature lines on automotive outer panels are difficult to make, measure, and inspect. This paper proposes an algorithm to fit a line-arc-line curve to the measured data points of a subtle feature line. First, based on an iterative method and random sampling consensus, the algorithm separates points corresponding to a circular arc. Further, the lines tangent to the circle are estimated based on the other data points. The algorithm repeats the procedure until the error is lower than the specified tolerance. The sensitivity and applicability of the algorithm were analyzed by applying it to simulated data points, experimental specimens, and actual automotive panels. The results demonstrated that the algorithm is robust to noise and surface waves and can be applied to the automotive panel-manufacturing process.

## 1. Introduction

### 1.1. Motiviations

**Figure 1:**
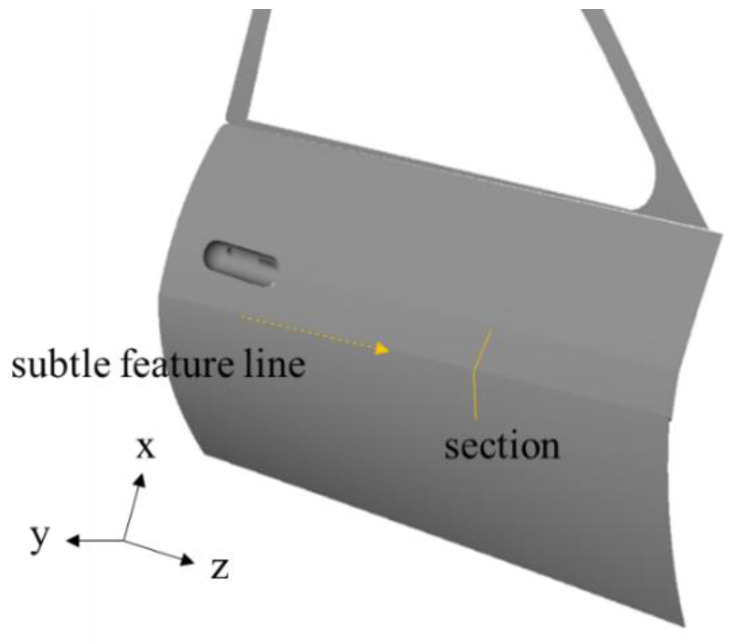
Subtle feature line on a door panel.

**Figure 2:**
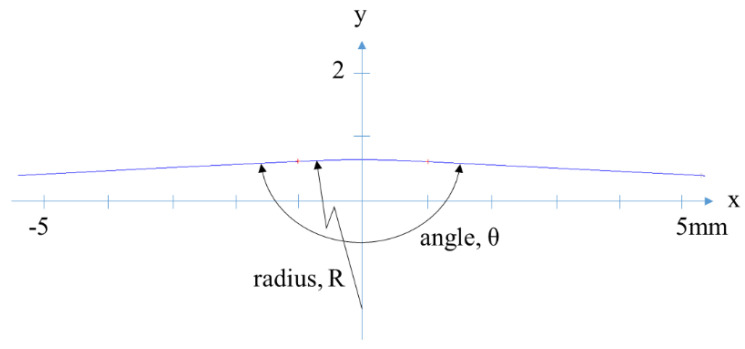
Sectional view of a subtle feature line.

The importance of automotive exteriors has grown as competition in the automotive industry has intensified. Automotive outer panels include narrow feature lines that exhibit long curved geometries [1], and the shape and dimensions of feature lines are essential because they determine the appearance of a car. Examples of feature lines on an automotive door outer panel and a sectional view are illustrated in Figure 1 and Figure 2, respectively. Automotive outer panels are manufactured using the stamping process, which is a metal-forming process that combines drawing and bending deformations. Therefore, it is difficult to form the panel into a designed shape. Feature lines with larger angles and smaller radii are more difficult to create and are known as subtle feature lines [2]. For example, an automaker defines a feature line with an angle greater than 155° and a radius less than 48 mm as a subtle feature line and manages it separately.

It is difficult to measure and inspect the shape of a subtle feature line because of the precision required and its small size. The subtle feature line section includes an arc with lines at either ends, as shown in Figure 2, and the radius (R) of the arc and angle (θ) between the two lines specifies the shape. With an increase and decrease in the angle and radius of the subtle feature line section, respectively, the width of the subtle features decreases, making the measurement and inspection difficult. Although the typical chord length of the arc in the section measures up to 10 mm, precision, measured in micrometers, is required to estimate the arc’s radius. In addition, the roughness of the sheet metal panel and noise in the measured data make inspecting subtle feature lines difficult.

### 1.2. State of the Art and Related Work

Template matching helps inspect the geometry of subtle features in automakers; however, it is not accurate and does not produce numerical results. There are two types of measurement methods: noncontact and contact methods [3]. A coordinate measuring machine (CMM) employs a contact method that moves a probe along the outer surface and measures its movement in three dimensions [4]. The measurement speed of the CMM is extremely low in contrast to other methods. A non-contact 3D scanner can quickly and simultaneously measure coordinates over a large area; however, its measurement accuracy is lower than contact methods. Typical methods are not suitable for measuring subtle features regarding the measurement scale and precision.

A confocal microscope [5] and surface-roughness testers can be used to measure the subtle feature line. Confocal microscopy, a non-contact mechanism, possesses high precision for small areas; however, it takes hours to measure a small region. A surface-roughness tester measures the surface’s roughness using a contact method and measures the surface profiles with sub-micrometer resolutions. However, surface-roughness testers are typically capable of measuring surface profiles up to a length of 10 mm. Lee et al. measured the sections of subtle feature lines using a surface-roughness tester and demonstrated that the tester is a good method for measuring sections of the subtle feature line [2]. This study used data points of the surface profiles measured using a surface-roughness tester.

It is difficult to estimate the angle and radius based on the measured points on a subtle feature line. As shown in Figure 2, the angle between the two lines and the radius of the central arc are the main parameters of the subtle feature line. The angles and radius can be inspected via the line-arc-line curve by approximating the measured points on a line-arc-line curve. In this study, the random sampling consensus (RANSAC) algorithm proposed by Fischler and Bolles [6] was used to approximate the measured points on a line-arc-line curve. RANSAC is an iterative method for estimating the parameters of a mathematical model from a dataset containing noise and outliers. This process includes two iterative steps. First, the algorithm randomly selects a sample subset of data from the input dataset and estimates the parameters of the model based on the sample data. Next, the algorithm checks the elements of the entire dataset that are consistent with the model obtained in the first step. The number of sample subsets was sufficiently small enough to determine the model parameters. If a data element does not fit the model within the tolerance limit, it is considered an outlier; otherwise, it is an inlier.

Chida and Masuda used the RANSAC algorithm to estimate the shapes from a measured point cloud [7]. They subdivided a point cloud into small regions to extract surfaces in practical time. Kawashima et al. used an algorithm to fit the line of a pipe using laser-scanned point clouds [8]. Robertson et al. applied the algorithm to classify discontinuities and extract parametric models from low-quality three-dimensional data [9]. This study used the RANSAC algorithm to estimate an arc based on a measured dataset. In this study, computation times were sufficiently quick because the number of data points was minor in contrast to the number of point clouds from the surface.

Fitting geometric shapes to point data is a common geometric problem. Many researchers studied fitting a geometric model to a point sequence or point cloud. Numerous techniques, such as least-squares, principal component analysis, optimal transport, graph theory, and machine learning, have been used for fitting geometric shapes to point data. Currently, circle fitting has become a trivial problem, as most algorithms are public and easily accessible over the internet. Bo et al. proposed a graph-based method to fit B-spline curves to a planar point cloud, in which multiple curves with intersections were extracted [10]. Suzuki et al. proposed a method for matching computer-aided design (CAD) data and scan data [11]. The scan data in their study were largely deformed simulation models, and the deformed CAD data were obtained using the matching method. He et al. [12] fitted B-spline curves to numerical control (NC) milling paths using the modified progressive and iterative approximation method proposed by Qi et al. [13]. They incorporated the energy term into a progressive iterative approximation method to avoid numerical instability and they lowered chord errors by stretching the energy term. 

Various fitting methods have been proposed for point data with noise and outliers [14,15,16,17]. Most of these methods first remove noise through clustering, thinning, or averaging; however, they often lose noteworthy features. Song proposed an algorithm to reconstruct curves from unorganized points using clustering, confining, and thinning. In this study, a minimal spanning tree is used to remove noise from the data [17]. Ghorbani and Khameneifar proposed an automatic reconstruction method for sectional airfoil profiles obtained from inspection data points [18]. They used a recursive weighted local least-squares technique to fit curves within the uncertainty measurement of data. The measured data in this study include wave and random noise, and the geometric model is known as a line-arc-line curve.

### 1.3. Contributions

This paper proposes a curve-fitting algorithm and analyzes its stability in practical applications. The algorithm fits a line-arc-line curve to the data points obtained by measuring the cross-section of the subtle feature lines using a surface-roughness tester. Three types of data points were used to analyze the stability of the algorithm: simulated data, measured data from laboratory specimens, and measured data from real panels. The main contributions of this paper are outlined as follows:

- A curve-fitting algorithm for evaluating the accuracy of the subtle feature line is presented.
- Numerical, experimental, and actual specimens are tested to verify the proposed algorithm.

Moreover some applications of the proposed algorithm are stated as follows:

- The algorithm can be applied to the automotive panel-manufacturing process.
- The stochastic method can be easily applicable to other fitting methods.

The rest of this paper is organized as follows: Section 2 provides details of the proposed algorithm. Section 3 describes the results and analysis of the algorithm. Finally, the last section concludes this paper.

## 2. Algorithm

**Figure 3:**
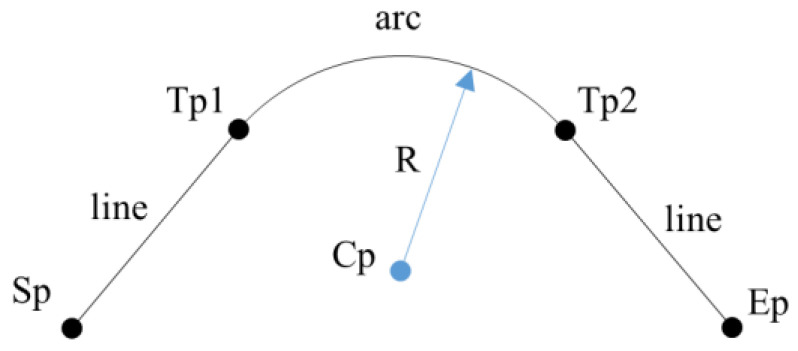
Line-arc-line curve model and parameters.

The algorithm comprises four iterative steps. First, the input data are separated into three groups. Second, the middle group is approximated as a circle. Third, two tangential lines on either side of the arc are estimated. Finally, the estimated model is evaluated. The algorithm repeats these four steps until the evaluation results are satisfactory. Figure 3 shows the line-arc-line curve model and its parameters to be estimated by the algorithm. The curve model is defined as an arc with two tangent line segments on either side. In the figure, Cp and R are the center and radius of the arc, respectively. The left line is specified by Sp and Tp1, and the right line is specified by Ep and Tp2. Tp1 and Tp2 are the tangent points between the arc and two lines. 

**Figure 4:**
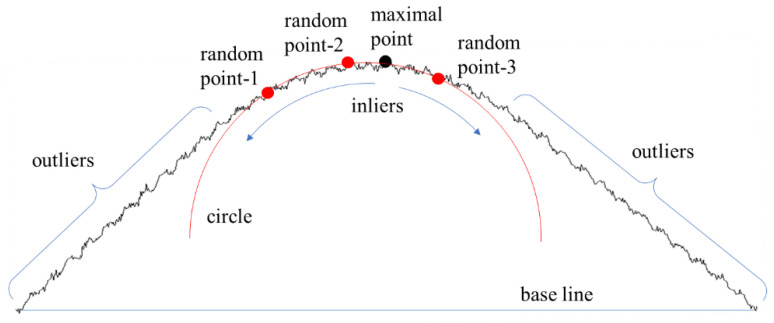
Outliers, inliers, and maximal point.

In the first step, the algorithm classifies the input dataset into three groups based on the RANSAC algorithm. Three points are randomly selected from the measured data, and these define a unique circle [19]. Subsequently, based on the circle, the algorithm separates the input data into three groups: left outliers, inliers, and right outliers. The inlier group is an arc segment, and the two outlier groups are line segments on either side of the arc. If the distance between the point and the circle is less than a specified tolerance, the point is classified as an inlier. In this study, the tolerance was set at five times the wave height. The inlier classification starts at the maximal point of the data and continues in both directions until the point is considered an outlier. Thus, the inlier group includes the maximal point, and each group defines a consecutive set of points in the input data. Figure 4 shows the three groups. The maximum data point is the point with the maximum perpendicular distance to the baseline connecting the first and last points of the data. The maximal point is selected from the arc segment because the arc segment is the only convex segment in subtle feature geometries.

The second step involves fitting a circle to the inlier points separated in the first step. In this study, Kasa’s algorithm [20] was adopted for the least-squares method of circle fitting. This algorithm minimizes the sum of the squared differences between the squared radii:

(1) $$\overset{N}{\underset{i=1}{\sum }}{\left(R_{i}^{2}-R^{2}\right)}^{2}$$

where $R_{i}=\sqrt{{\left(x_{i}-a\right)}^{2}+{\left(y_{i}-b\right)}^{2}}$. $\left(x_{i},\;y_{i}\right)$ represents the *i*-th data point, and *R* and (*a*, *b*) are the radius and center of the circle, respectively. The output of this step is the center and radius of the arc in a subtle feature line. The starting and end points of the arc are determined in the next step.

In the third step, the two linear segments tangent to the circle fitted in the second step are estimated. The geometric center of the points at the end of the outliers is used as the starting point of the line. The number of points in the geometric center was determined by considering the noise and waviness of the data. Because the noise was significantly smaller than the wave height, waviness was considered in this study. These points included more than three waves. The starting point and tangential continuity conditions determine the unique line. Given the starting point of the line (**S**p), center point (**C**p), and radius (R) of the circle, the tangent point (**T**p) is calculated as follows:

(2) Tp=Cp−m U ±h V, 

where

$$m=\frac{R^{2}}{\left|Cp-Sp\right|}$$

$$h=\sqrt{R^{2}-m^{2}}$$

$$U=\frac{Cp-Sp}{\left|Cp-Sp\right|}$$

$$V=\left(U_{y},-U_{x}\right).$$

The endpoint (**E**p) and **T**p of the right line can be calculated in a manner similar to that of the left line.

Now we have all parameters of the subtle feature line defined by the line-arc-line curve. As shown in Figure 3, the two tangent points specify the end limits of the arc segment in the subtle feature line’s geometry. The fitted model is approximated because the data points include both noise and waves. Therefore, in the final step, an evaluation of the estimated model is required. The estimated model is evaluated using the root-mean-square error (RMSE) as follows:

(3) $$RMSE=\sqrt{\frac{1}{n}\overset{n}{\underset{i=1}{\sum }}e_{i}^{2}}\;,\;$$

where $e_{i}$ represents the distance between the *i*-th point and estimated line-arc-line curve, with *n* being the number of data points. The distance between a point and a line or arc can easily be calculated. The algorithm repeats until the RMSE is less than the specified allowance and stops when the number of iterations reaches a predetermined maximum.

## 3. Results and Analysis

### 3.1. Numerical Experiments

A numerical experiment was conducted to verify the proposed algorithm. The arc radius and angle of the subtle feature line and the noise of the simulated data points were used as the experimental variables. The focus of the experimental analysis was the deviations observed in the arc’s radius and angle of the estimated geometry. In all experiments, we sampled 800 points at intervals of 0.005 mm based on a subtle feature curve symmetric about the y-axis. The x-coordinate values of the data points ranged from −2.0 to +2.0 mm. Because the algorithm is based on a stochastic process, the estimation results may vary for every trial. However, the estimation accuracy was very high, and the differences between the different experiments were very small. The estimates of the following experiments are the results of one trial.

**Figure 5:**
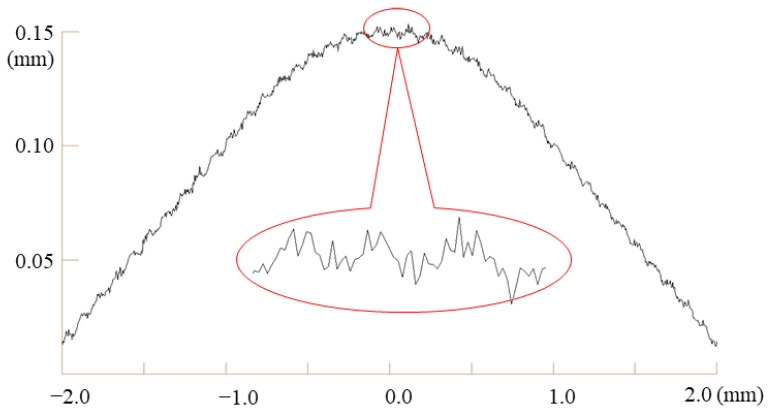
Simulated data points with an arc radius of 10 mm and angle of 170°. The mean and standard deviation of the Gaussian noise are 0 and 0.001 mm, respectively. The height and length of the wave noise are 0.003 and 0.1 mm, respectively.

In the first experiment, we added two types of noise (roughness and waviness) to the y-coordinate values of the sampled points. Figure 5 shows the noise types of the data points sampled from a subtle feature curve. The roughness was simulated using an independent Gaussian (also known as a normal distribution) noise, and the waviness was simulated using a sine curve. The noise parameters were defined as follows:

- Mean of the Gaussian noise = 0. mm;
- Standard deviation of the Gaussian noise = 0.001 mm;
- Wavelength of a sine curve = 0.1 mm;
- Wave height of a sine curve = 0.003 mm.

Table 1 lists the results for different feature curve angles with an arc radius of 10 mm. The angles were accurately estimated; however, as the angle increased, the estimated arc radius decreased, and the error increased. The error rate was calculated as follows.

(4) $$Error\;rate=\frac{\left(true\;value\right)-\left(estimated\;value\right)}{\left(true\;value\right)}\times 100\%.\;$$

In the second experiment, the effects of the different radii were analyzed. Table 2 lists the results for different feature curve radii at an angle of 170°. All other variables were the same as those used in the first experiment, as listed in Table 1. Similarly to the first experiment, the angles were accurately estimated for all cases. As the input radius increases, the estimated radius gradually increases as well. At a radius of 5 mm, the estimated radius was smaller than the given value; however, at 20 mm, it was greater than the given value. As listed in Table 1 and Table 2, the RMSE values were similar because the noise levels were identical.

Moreover, sensitivities relative to different noise types were analyzed. Table 3, Table 4 and Table 5 list these results. In all cases, the same feature curve was used, with an arc radius of 10 mm and an angle of 170°. The default standard deviation of Gaussian noise was 0.001 mm, and the default height and length of the sine wave were 0.003 and 0.1 mm, respectively. In the tables, the asterisk represents the default parameter value. As shown in the tables, the angles were accurately estimated for all cases. However, as noise deviation and height increased, the error in the estimated radius also increased (Table 3 and Table 4). As listed in Table 5, the wavelength of the sine wave noise is variable, while other parameters have default values. This demonstrates that the longer the wavelength of the noise, the more accurate the estimation of the radius. As listed in the tables, except for the wavelength, the Gaussian noise and wave height affected the RMSE.

### 3.2. Application of Specimen Panels to Metal Forming Studies

**Figure 6:**
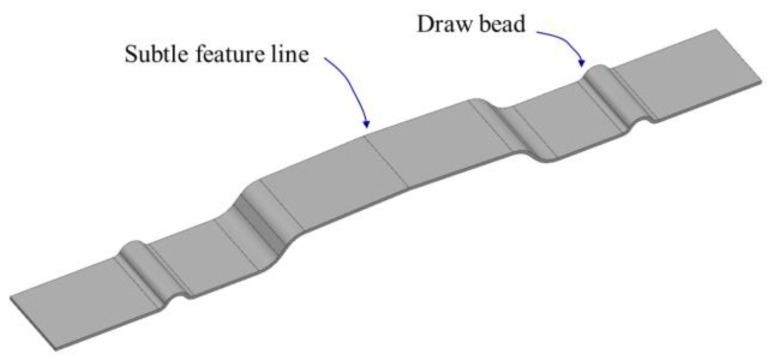
Experimental model with a subtle feature line in the center.

To accurately make subtle feature lines for studies on sheet metal formation, several experimental samples were prepared and measured in laboratories. A consistent measurement method in these studies is important to obtain reliable results. The proposed algorithm was applied to the measured data points. Lee et al. [2] proposed a simple geometry, including the subtle feature line shown in Figure 6. The geometry was symmetrical with a subtle feature line in the center, and beads were drawn on either side to control the flow of the material. While the draw beads hold the material from the sides, the punch operation pushes the material, resulting in tensile and bending deformations in the center region. This manufacturing process mimics the stamping and sheet-metal forming processes of automotive panels.

**Figure 7:**
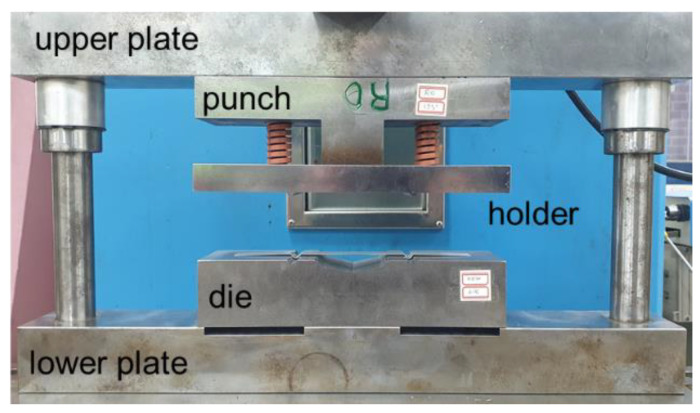
Die set to study the formation process of subtle feature lines.

Figure 7 shows a die set used in the laboratory to study the formation process of subtle feature lines. Because a universal testing machine cannot generate an adequate blank holding force in one stroke, we used a two-stage manufacturing process. In the first stage, draw beads formed on both sides using a holder without a punch. In the second stage, the holder applied pressure to the draw beads. The force on the draw beads increased as the punch was lowered. Due to the friction and geometry of the draw beads, the material was held in the die set. As the punch moves, tensile and bending deformations occur in the middle section, and the final geometry is produced.

In the formation experiments, the upper plate of the die set was joined using a mechanical press (Figure 7). The distance between the beads was 90 mm. In the first stage of the draw-bead formation, the holder was fixed to the upper plate. In the second stage of subtle feature line formation, a spring with a stiffness of 158 N/mm and a length of 65 mm was used. Punches and dies of different angles and radii were used in the experiments. 

**Figure 8:**
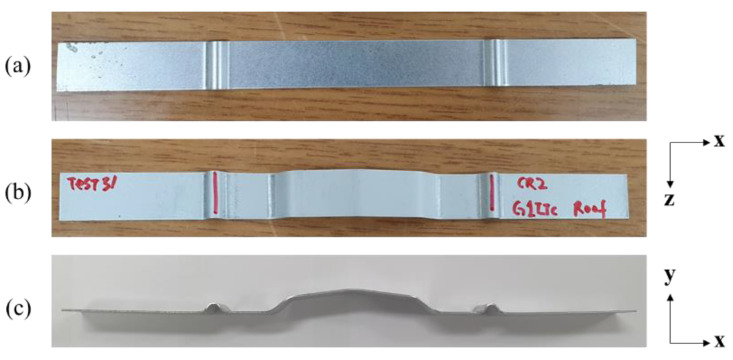
Fabricated specimen: (**a**) after the bead-forming process, (**b**) top view after the final formation process, and (**c**) side view.

**Figure 9:**
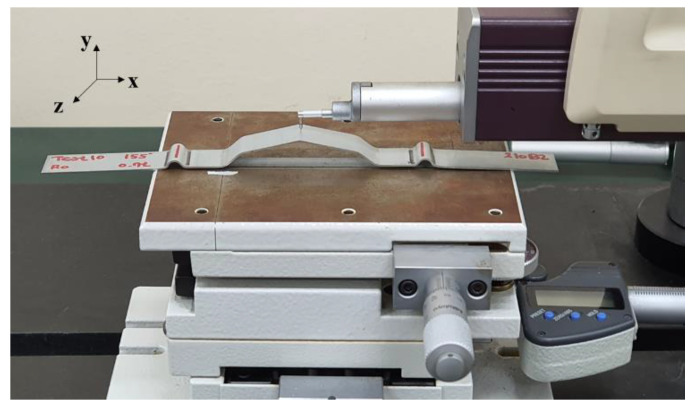
Surface profile measuring using a roughness tester (Mitutoyo SJ-400).

Figure 8 illustrates the two-stage manufacturing process. The sheet shown in Figure 8a is the result of the bead-forming process. Figure 8b shows the result of the final formation process, and Figure 8c shows the side view of the final sheet. The angle of the punch was 155°, and the radius of the punch was 0 mm. The material used was drawing-quality mild steel (CR2) with a thickness of 0.7 mm. The surface profile of the manufactured specimen was measured using a surface-roughness tester (SJ-400, Mitutoyo) (Figure 9). The measured length was 4 mm, and the point spacing was 0.5 µm; therefore, the number of data points was 8000. Three punch angles (155°, 165°, and 170°) were tested under the same conditions. As a result of visual analysis, the proposed algorithm fits the line-arc-line curve to the measured subtle features of the specimen panels. The RMSEs were 1.075, 1.136, and 2.189 µm for specimens with punch angles of 155°, 165°, and 170°, respectively.

### 3.3. Application of Actual Automotive Panels

**Figure 10:**
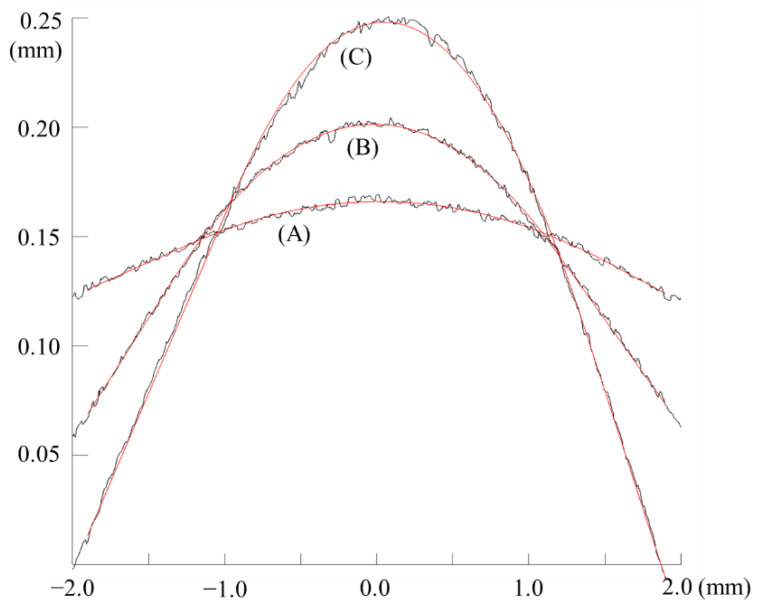
Fitted curves and measured data points of the subtle features on automobile outer panels. The values for the cases, (A), (B) and (C) are given in Table 6.

The proposed algorithm was applied to data points measured on real automotive outer panels. A column-mounted surface-roughness tester (SJ-400, Mitutoyo) was used to measure the profiles of subtle features. The measured length was 4 mm and the point spacing was 0.5 µm. The number of data points was 8000. Figure 10 shows the estimated feature curves overlaid on the measured profiles. From the visual analysis, the proposed algorithm effectively fits the line-arc-line curves to the measured subtle features. In all cases, the RMSEs were <3 µm. As listed in Table 6, the measured results were compared with the design values of automotive panels. Due to the nature of sheet metal forming, the measured angle and radius are larger than the design values.

## 4. Conclusions

This study proposed an algorithm to fit a line-arc-line curve to the measured data and estimate the radius and angle of a subtle feature line. The proposed algorithm is based on the RANSAC method and tangential continuity condition. Kasa’s algorithm was used in this study because the implementation of the proposed algorithm requires a circle fitting method. However, other circle-fitting methods can also be used. The results demonstrate that the proposed algorithm is stable against noise with respect to the measured data. The radius was estimated with less than 1% error, and the angle was estimated with less than 0.3% error in most simulated experiments. Using this algorithm, consistent curvature estimation can easily be implemented in the analysis of subtle feature lines and can be applied to the automotive panel-manufacturing process. Although it is not easy to measure and acquire point data in the manufacturing process, it is expected that the proposed concept can easily be applied to other fitting methods.

## Figures and Tables

**Table 1:** Estimated angles and radii for different angles with an arc radius of 10 mm.

True Angle (°)	160.0	165.0	170.0	175.0
Estimated Angle (°)	159.9	165.0	170.0	175.0
(Error Rate)	(−0.06%)	(0.00%)	(0.00%)	(0.00%)
Estimated R (mm)	10.01	10.00	9.97	9.70
(Error Rate)	(0.10%)	(0.00%)	(−0.30%)	(−3.00%)
RMSE (µm)	1.48	1.48	1.48	1.49

**Table 2:** Estimated arc radii and angles for different radii with an angle of 170°.

True R (mm)	5.00	10.00	15.00	20.00
Estimated R (mm)	4.97	9.97	15.01	20.04
(Error Rate)	(−0.60%)	(−0.30%)	(0.07%)	(0.20%)
Estimated Angle (°)	170.0	170.0	170.0	169.9
(Error Rate)	(0.00%)	(0.00%)	(0.00%)	(−0.06%)
RMSE (µm)	1.48	1.48	1.48	1.48

**Table 3:** Estimated radii and angles for different standard deviations of the Gaussian noise. Reference value is marked with an asterisk (*).

Standard Deviation (mm)	0.0001	0.001 *	0.002	0.003
Estimated R (mm)	9.98	9.97	9.95	9.89
(Error Rate)	(−0.20%)	(−0.30%)	(−0.50%)	(−1.10%)
Estimated Angle (°)	170.0	170.0	170.0	170.0
(Error Rate)	(0.00%)	(0.00%)	(0.00%)	(0.00%)
RMSE (µm)	1.07	1.48	2.29	3.20

**Table 4:** Estimated radii and angles for different heights of the sine wave. Reference value is marked with an asterisk (*).

Wave Height (mm)	0.003 *	0.005	0.01	0.02
Estimated R (mm)	9.97	9.95	9.84	9.72
(Error Rate)	(−0.30%)	(−0.50%)	(−1.60%)	(−2.80%)
Estimated Angle (°)	170.0	170.0	170.0	169.9
(Error Rate)	(0.00%)	(0.00%)	(0.00%)	(−0.06%)
RMSE (µm)	1.48	2.06	3.72	7.23

**Table 5:** Estimated radii and angles for different wavelengths of the sine wave. Reference value is marked with an asterisk (*).

Wavelength (mm)	0.05	0.1 *	0.2	0.5
Estimated R (mm)	9.96	9.97	9.98	9.98
(Error Rate)	(−0.40%)	(−0.30%)	(−0.20%)	(−0.20%)
Estimated Angle (°)	170.0	170.0	170.0	170.0
(Error Rate)	(0.00%)	(0.00%)	(0.00%)	(0.00%)
RMSE (µm)	1.46	1.48	1.42	1.45

**Table 6:** Comparison between design and measured values. The three cases, (A), (B) and (C) are graphically presented in Figure 10.

		**(A** **)**	**(B** **)**	**(C** **)**
Design	Angle (degree)	173.4	167.2	155.2
	Radius (mm)	25.65	8.00	5.45
Measured	Angle (degree)	176.1	168.2	158.7
	Radius (mm)	40.38	12.33	6.26
	RMSE (µm)	1.27	1.27	2.33

## Data Availability

Data available upon request due to restrictions. The data presented in this study are available upon request from the corresponding author. The data are not publicly available due to grant and patent restrictions.
